# Long-term functional and clinical outcomes of intraarticular double-cross-linked high molecular weight hyaluronic acid (Crespine Gel Plus) injection in knee osteoarthritis: a one year prospective study

**DOI:** 10.1186/s12891-026-09588-1

**Published:** 2026-02-09

**Authors:** Vijaya Kumar L. Suppan, Mei Mei Tew, Hrishinilaavenn Muthusamy, Jin May Voon, Nik Abdul Muhaimin Nik Ramli, Aaron Siow Jon Phuah, Dinesh Murugaiah

**Affiliations:** 1 Department of Orthopaedics, Sultan Abdul Halim Hospital, Sungai Petani, 08000 Kedah, Malaysia; 2 Clinical Research Centre, Sultan Abdul Halim Hospital, Institute for Clinical Research, National Institute of Health, Ministry of Health Malaysia, 08000 Kedah, Malaysia

**Keywords:** Osteoarthritis, Knee, Hyaluronic Acid, Injections, Intra-Articular, Patient-Reported Outcome Measures, Treatment Outcome

## Abstract

**Background:**

Knee osteoarthritis (OA) causes substantial pain and disability. Double cross-linked, high-molecular-weight hyaluronic acid (HMWHA; Crespine Gel Plus) may extend symptom relief beyond the typical 3 to 6 month horizon, but robust 12-month data remain limited. This study aimed to evaluate short- and long-term outcomes over 12 months following a single Crespine Gel Plus injection in routine care.

**Methods:**

Prospective single arm cohort at a Malaysian Hospital from June 2023 to June 2025. Adults with radiographically confirmed knee OA (Kellgren–Lawrence [KL] grades I–III, or IV if declining surgery) received a single 2 mL intra-articular injection. Outcomes were KOOS4 (primary; 0–100, higher = better) and VAS pain (0–10; higher worst) along with KOOS4 Subscales. Assessments were at baseline, 3 months, and 12 months. Linear mixed-effects models (participant random intercepts; REML; Satterthwaite dfs) adjusted for age, gender, BMI, and KL grade.

**Results:**

Of 111 participants (mean age 65.1 ± 7.8; 66.6% female; BMI 28.2 ± 4.9 kg/m2), 107 (96.4%) completed 3-month follow up and 92 (82.9%) completed 12-month follow up. Adjusted KOOS4 improved by 8.59 points (95% CI 5.48–11.69) at 3 months and 8.78 (5.51–12.05) at 12 months (both *p* < 0.001), exceeding customary minimal clinically important differences (MCIDs). VAS decreased by 1.68 points (-2.03 to -1.33) at 3 months and 1.64 points (-2.01 to -1.27) at 12 months (both *p* < 0.001). KOOS Pain, Symptoms, ADL, and QoL improved at at 3 and 12 months, with small, non-significant changes between 3 and 12 months. Higher BMI predicted worse adjusted KOOS outcomes; age and KL grade were not significant predictors. No major adverse events occurred.

**Conclusion:**

A single injection Crespine Gel Plus was associated with clinically meaningful improvements in pain and function that emerged by 3 months and were maintained to 12 months, with a favourable tolerability profile.

## Introduction

Knee osteoarthritis (OA) is the most prevalent form of arthritis and a major driver of chronic pain, disability, and health-care utilization worldwide. The pathological features of knee osteoarthritis including progressive cartilage loss, subchondral bone remodeling, osteophyte formation, and synovial inflammation, which in turn translate into pain, stiffness, impaired function, and ultimately diminished quality of life [1, 2] The public-health burden of knee OA is substantial and rising with aging populations, increasing obesity, and sedentary lifestyles. The Global Burden of Disease 2021 analysis estimates that more than 250 million people live with knee OA, with further growth expected over coming decades [3].

In Southeast Asia, including Malaysia, demographic and cultural factors amplify this burden, whereby many patients defer arthroplasty due to preference, access, or financial reasons. Therefore, most patient remain on conservative care pathways for prolonged periods [4–6]. In this context, scalable, durable non-surgical interventions are needed to sustain symptom relief and maintain function while surgery is delayed or optimized. Among nonoperative options, intra-articular hyaluronic acid (IAHA) is widely used for symptomatic knee OA. IAHA aims to restore the viscoelastic properties of synovial fluid, potentially reducing friction, improving shock absorption, and modulating intra-articular inflammation. Randomized and observational studies show IAHA brings clinically relevant pain reduction that often peaks around 8 weeks and can persist to approximately 6 months; in several comparisons IAHA outperforms standard oral analgesics [7, 8] Published studies suggest that exposure to IAHA is associated with delayed time to total knee replacement (TKR) by roughly 2 to 3.6 years in some cohorts, indicating possible disease-trajectory modification at the population level [9–13].

Nevertheless, not all HA products are the same, they differed by formulation characteristics, including molecular weight, concentration, and cross-linking, which may affect its intra-articular residence time, viscoelastic behaviour, and susceptibility to enzymatic degradation. Evidence indicates that high-molecular-weight (HMW) HA preparations tend to produce larger clinical effects and lower discontinuation rates than low-molecular-weight (LMW) HA formulations [1, 9, 13, 14]. Regimens also vary, some products are administered as single injection, whereas others are given in multi-injection series (e.g., 3–5 weekly doses). These differences may influence durability of benefit, patient convenience, cost, and safety profiles [8, 13–16].

Crespine Gel Plus is a double cross-linked high-molecular-weight hyaluronic acid engineered to increase intra-articular durability through enhanced viscoelasticity and resistance to hyaluronidase-mediated degradation, these features intended to extend symptom control with a single 2-mL injection. Published evidence specific to Crespine has, to date, been limited to ≤ 9 months, demonstrating sustained improvements in pain and function within that window [17]. More broadly, most IAHA trials emphasize outcomes ≤ 6 months, leaving longer-term durability, particularly with single-episode, cross-linked HMW formulations which are less well characterized [8, 13–16, 18]. To address this gap, a prospective observational study is carried out to evaluate longer term outcome of a single intra-articular injection of Crespine Gel Plus in patients with symptomatic knee osteoarthritis. The primary aim of this study was to evaluate short- and long-term (12-month) changes in pain and function following a single intra-articular injection of double cross-linked high-molecular-weight hyaluronic acid (Crespine Gel Plus) in patients with symptomatic knee osteoarthritis in routine clinical practice. Secondary aims were to assess changes in individual KOOS subscales and VAS pain scores and evaluate safety over one year.

## Methods

### Study design and setting

We conducted a prospective observational study at Sultan Abdul Halim Hospital (HSAH), Kedah, Malaysia, from June 2023 to June 2025. The protocol was approved by the Medical Research and Ethics Committee (MREC) (NMRR ID-23–01219-VFL), Ministry of Health Malaysia. All procedures adhered to the Declaration of Helsinki, and written informed consent was obtained from all participants.

### Participants

Eligible patients were ambulatory adults aged 40–80 years with radiographically confirmed OA in one or both knees. Radiographic severity was Kellgren–Lawrence (KL) grade I–III, or grade IV in patients who had declined surgery, documented on standard knee radiographs within 6 months prior to enrolment and received a single 2 mL intra-articular injection of double cross-linked high-molecular-weight hyaluronic acid (Crespine Gel Plus) to the index knee using standard aseptic technique. Key exclusions were participation in another clinical trial within 90 days; any IAHA injection to the index knee within 120 days; severe hip OA; known hypersensitivity to hyaluronic acid; significant systemic illness; planned total knee replacement at the target joint; bleeding disorder or coagulation defect; history of stroke with major neurological deficit; neuropathic pain or sensory disorder; prior knee infection (septic arthritis or tuberculosis); active skin disorder or infection overlying the index knee; or inability/unwillingness to provide consent or comply with study procedures.

In routine clinical practice, intra-articular hyaluronic acid injection was offered based on clinical indication rather than radiographic severity alone. For patients with Kellgren–Lawrence (KL) grade I–III knee osteoarthritis, treatment was considered in the presence of persistent knee pain and functional limitation that were inadequately controlled with initial conservative measures, such as oral analgesics, activity modification, and/or physiotherapy. Intra-articular hyaluronic acid was not used as first-line therapy but as part of a stepwise conservative management approach, particularly in older patients or in those for whom prolonged use of oral analgesics or non-steroidal anti-inflammatory drugs was considered undesirable due to potential adverse effects. For patients with KL grade IV disease, injection therapy was considered when patients declined, were unsuitable for, or wished to defer surgical intervention.

### Injection procedure and post-injection care

All injections were performed by experienced orthopaedic surgeons under aseptic conditions using a superolateral approach with the knee in slight flexion, guided by anatomical landmarks (superolateral patellar border and femoral condyle). A single 2-mL dose of Crespine Gel Plus was injected intra-articularly. Post-injection care included brief observation, avoidance of strenuous activity, and use of ice for mild discomfort.

### Outcomes measures

Functional outcome measures, including the KOOS and VAS, were primarily self-administered by patients. Assistance from trained research personnel was provided when required (e.g., for clarification or literacy support), following standardized instructions and without interpretation or influence on patient responses.

The primary outcome was the Knee Injury and Osteoarthritis Outcome Score (KOOS4). KOOS contains 5 domains, namely, Pain, Symptoms, Activities of Daily Living (ADL), Sport and Recreation Function (Sport/Rec) and knee related Quality of Life (QoL). KOOS4 is defined as the arithmetic mean of KOOS Pain, Symptoms, ADL, and QoL subscales (0–100; higher scores indicate better status). The Sport/Recreation subscale was excluded due to limited applicability in older, lower-activity cohorts and its propensity for floor effects and structural missingness, which can bias composite estimates; use of KOOS4 is consistent with prior methodological guidance and applications [19–22].

Secondary outcomes were VAS pain (0–10; higher scores indicate worse pain) and each KOOS4 subscale (Pain, Symptoms, ADL, QoL) analyzed individually. Safety endpoints included the incidence, type, and severity of adverse events (local injection-site reactions and systemic events). Outcome measures were collected at baseline, 3 months, and 12 months post-injection. To facilitate clinical interpretation, this study referenced minimal clinically important differences (MCIDs) of 1–2 points for VAS pain and 8–10 points for KOOS subscales/KOOS4 [23–25]. Any adverse events were collected throughout the study.

### Concomitant therapies

This study was observational and evaluated outcomes following routine clinical practice, with no study-mandated interventions. The exposure of interest was a single intra-articular injection of double cross-linked high-molecular-weight hyaluronic acid (Crespine Gel Plus), administered as part of usual care. No standardized or supervised exercise therapy program was prescribed within the study protocol. Patients continued to receive usual clinical management at the discretion of the treating clinician, which could include general advice on muscle strengthening, knee range-of-motion exercises, weight management, and activity modification. Referral for formal physiotherapy was based on individual clinical judgement and was not standardized. Concomitant therapies, including rescue analgesic use and physiotherapy, were recorded during follow-up, and are reported descriptively; no formal adjustment for changes in co-interventions was performed.

### Sample size

Sample size was estimated using a conservative two-means framework based on prior data (pre-injection mean KOOS 51.9 [SD 15.3] vs post-injection mean KOOS 59.2 [SD 14.3]). With two-sided α = 0.01 and 90% power, the required sample was 87 participants; allowing for 20% drop out, the final required sample size was 109. Although the present study uses repeated measures, this approach was adopted as a conservative planning strategy [26–28].

### Statistical analysis

Longitudinal changes were analyzed using linear mixed-effects models (LMMs) with random intercepts for participants, Restricted (or Residual) Maximum Likelihood (REML) estimation, and Satterthwaite approximations for denominator degrees of freedom. Fixed effects included time (baseline, 3 months, 12 months), KL grade, and gender (categorical), with age and BMI as continuous covariates. Model-based estimated marginal means and 95% confidence intervals were reported, with pairwise comparison for baseline to 3 months and baseline to 12 months; changes are expressed as positive values for KOOS (improvement) and negative values for VAS (improvement). Missing data were assumed to be missing at random (MAR), and longitudinal analyses were performed using linear mixed-effects models, which incorporate all available observations without imputation and provide unbiased estimates under the MAR assumption. Analyses were performed in IBM SPSS Statistics version 31.

## Results

**Fig. 1:**
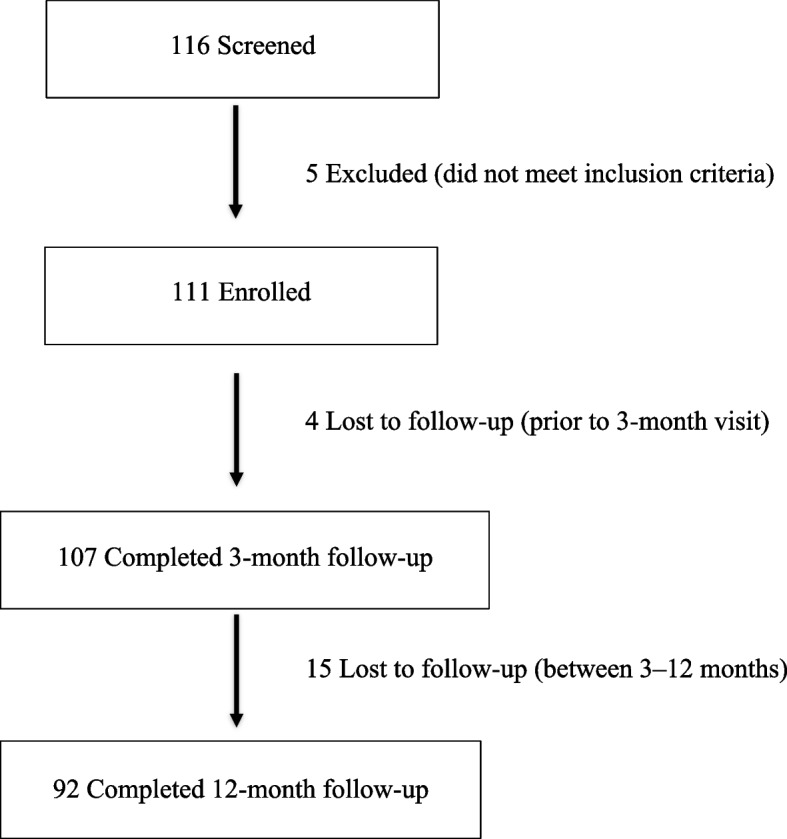
Subject flow diagram: subject flow from screening to 12-month follow-up

Of 116 screened patients, 5 (4.3%) were excluded for ineligibility and 111 were enrolled. By 3 months, 4/111 (3.6%) were lost to follow-up, yielding 107/111 (96.4%) with 3-month data; between 3 and 12 months, a further 15/107 (14.0%) were lost, resulting in 92/111 (82.9%) completing 12 months (Fig. 1). Among the 111 participants, 37 (33.3%) were male and 74 (66.7%) female; mean age was 65.1 ± 7.8 years and mean BMI 28.2 ± 4.8 kg/m^2^ (underweight 1.8%, normal 23.4%, overweight 41.4%, obese 33.3%). The affected knee was bilateral in 97 (87.4%), and mean OA duration was 6.7 ± 4.6 years. Kellgren–Lawrence grading at baseline was: grade I, 3 (2.7%); grade II, 44 (39.6%); grade III, 56 (50.5%); and grade IV, 8 (7.2%) (Table 1).

**Table 1:** Baseline characteristics of study participants (*N* = 111)

Variable	n (%)	Mean ± SD
Gender		
Male	37 (33.3)	
Female	74 (66.7)	
Race		
Malay	52 (46.8)	
Chinese	31 (27.9)	
India	28 (25.2)	
Age (years)		65.1 ± 7.8
BMI (kg/m^2^)		28.2 ± 4.8
Underweight (< 18.5)	2 (1.8)	
Normal (18.5–24.9)	26 (23.4)	
Overweight (25.0–29.9)	46 (41.4)	
Obese (≥ 30.0)	37 (33.3)	
Affected Knee		
Left	5 (4.5)	
Right	9 (8.1)	
Bilateral	97 (87.4)	
OA History (years)		6.7 ± 4.6
KL grade		
Grade 1	3 (2.7)	
Grade 2	44 (39.6)	
Grade 3	56 (50.5)	
Grade 4	8 (7.2)	

*Abbreviations: BMI* Body Mass Index, *OA* Osteoarthritis, *KL* Kellgren–Lawrence

Given the single-arm observational design, changes in outcomes over time are reported as associations following the intervention, rather than evidence of causal effects. Adjusted means improved from baseline to 3 months and were largely maintained at 12 months (Table 2). KOOS4 rose from 62.2 (SE 2.50) at baseline to 70.8 (SE 2.51) at 3 months and 71.0 (SE 2.57) at 12 months (*p* < 0.001 for time). Pairwise comparison (Table 3) showed 8.59 points (95% CI 5.48–11.69) at 3 months and 8.78 points (95% CI 5.51–12.05) at 12 months (both *p* < 0.001), exceeding customary KOOS MCID (8–10 points). The 3 to 12-month change for KOOS4 was 0.19 (95% CI—3.47 to 3.09, *p* = 0.907), indicating sustainability of the improvement at 3 months.

**Table 2:** Adjusted estimated marginal means over time (REML LMM)*

Outcome^**†**^	Baseline, Mean (SE)	3 months Mean (SE)	12 months Mean (SE)	*p*-value
KOOS4 (primary; higher = better)	62.2 (2.50)	70.8 (2.51)	71.0 (2.57)	< 0.001
KOOS Pain	67.2 (2.83)	76.9 (2.85)	74.8 (2.92)	< 0.001
KOOS Symptoms	72.1 (2.72)	79.7 (2.74)	78.7 (2.81)	< 0.001
KOOS ADL	63.3 (2.67)	74.2 (2.70)	74.2 (2.78)	< 0.001
KOOS QoL	45.7 (3.06)	52.0 (3.07)	56.4 (3.15)	< 0.001
VAS (0–10, higher = worse)	4.80 (0.27)	3.12 (0.27)	3.17 (0.28)	< 0.001

**Table 3:** Adjusted pairwise changes from baseline (REML LMM)*

Outcome^**†**^	3 months Mean difference (95% CI)	*p*-value	12 months Mean difference (95% CI)	*p*-value	3 months vs 12 months Mean difference (95% CI)	*p*-value
KOOS4	8.59 (5.48 to 11.69)	< 0.001	8.78 (5.51 to 12.05)	< 0.001	0.19 (−3.47 to 3.09)	0.907
KOOS Pain	9.75 (6.10 to 13.40)	< 0.001	7.64 (3.80 to 11.48)	< 0.001	−2.11 (−5.97 to 1.75)	0.282
KOOS Symptoms	7.64 (4.00 to 11.27)	< 0.001	6.66 (2.84 to 10.48)	< 0.001	−0.97 (−4.81 to 2.87)	0.617
KOOS ADL	10.55 (7.00 to 14.14)	< 0.001	10.17 (6.40 to 13.95)	< 0.001	0.07 (−4.8 to 4.63)	0.848
KOOS QoL	6.29 (2.27 to 10.32)	< 0.001	10.62 (6.39 to 14.85)	< 0.001	4.33 (0.73 to 8.58)	0.046
VAS (0–10)	−1.68 (−2.03 to −1.33)	< 0.001	−1.64 (−2.01 to −1.27)	< 0.001	0.04 (−0.42 to 0.33)	0.813

^*^Linear mixed-effects models fit via REML with Satterthwaite df, random intercept for participant, identity residual covariance for repeated time (baseline, 3, 12 months). Fixed effects: time (categorical), gender, Kellgren–Lawrence (KL) grade; covariates: BMI and age (entered as continuous). Estimated marginal means (and pairwise contrasts) are adjusted for gender and KL grade and evaluated at the sample means of BMI (≈28.2 kg/m^2^) and age (≈65.1 years). Pairwise comparisons used Least Significant Difference (LSD; no multiplicity adjustment); two-sided α = 0.05

^†^Outcomes: KOOS4 = mean of KOOS Pain, Symptoms, ADL, and QoL (0–100; higher = better). VAS = Visual Analog Scale pain (0–10; higher = worse)

For KOOS sub-domains, KOOS Pain improved 9.75 points (95% CI 6.10–13.40, *p* < 0.001) at 3 months and 7.64 points (95% CI 3.80–11.48, *p* < 0.001) at 12 months; KOOS Symptoms improved 7.64 points (95% CI 4.00–11.27, *p* < 0.001) and 6.66 points (95% CI 2.84–10.48, *p* < 0.001); KOOS ADL improved 10.55 points (95% CI 7.00–14.14, *p* < 0.001) and 10.17 points (95% CI 6.40–13.95, *p* < 0.001). KOOS QoL improved 6.29 points (95% CI 2.27–10.32, *p* < 0.001) at 3 months and 10.62 points (95% CI 6.86–14.38, *p* < 0.001) at 12 months, with a small but statistically significant additional gain from 3 to 12 months (4.33, 95% CI 0.73–8.58, *p* = 0.046). Improvements on KOOS4 and most subscales met or exceeded MCID at both 3 and 12 months, with minimal change thereafter, consistent with sustainable benefit through one year; QoL continued to accrue modest gains between 3 and 12 months (Tables 2 and 3). For KOOS QoL, although the incremental improvement between 3 and 12 months reached statistical significance, MCID thresholds are primarily defined for change from baseline rather than between follow-up visits. Notably, the improvement from baseline to 12 months exceeded established MCID criteria, indicating clinically meaningful long-term benefit.

For pain assessment using Visual Analog Scale (VAS, 0–10; higher = worse), the VAS decreased from 4.80 (SE 0.27) at baseline to 3.12 (SE 0.27) at 3 months and 3.17 (SE 0.28) at 12 months (*p* < 0.001). Baseline contrasts showed −1.68 (95% CI −2.03 to −1.33, *p* < 0.001) at 3 months and −1.64 (95% CI −2.01 to −1.27, *p* < 0.001) at 12 months, both exceeding the VAS MCID (1–2 points). The 3 to 12-month change was 0.04 (95% CI −0.42 to 0.33, *p* = 0.813); between 3 and 12 months, VAS pain scores showed no statistically significant change, indicating stable pain relief over time (Table 3).

In the LMMs, time effect was significant for all endpoints (all *p* < 0.001). BMI was an independent negative predictor across KOOS outcomes (e.g., KOOS4 β = −0.948 points per kg/m^2^ (95% CI −1.541 to −0.355; *p* = 0.002); with similar effects for Pain, Symptoms, ADL, and QoL (all *p* ≤ 0.05); expressed per 5 kg/m^2^, this corresponds to around—4.7 to −5.4 KOOS points. For VAS, the BMI association was small and not significant (β = + 0.055 per kg/m^2^; 95% CI −0.008 to 0.119; p = 0.087). Age, gender, and KL grade showed no overall effects on KOOS4, Symptoms, QoL, or VAS (all *p* > 0.05), with two exceptions confined to ADL; a modest negative slope for age (β = −0.393 per year; *p* = 0.047) and higher scores in male (β = 6.558; *p* = 0.022) (Table 4).

**Table 4:** Covariate effects from linear mixed-effects models (REML; random intercepts)

Outcome (higher = better, except VAS)	BMI β per kg/m^2^ (95% CI)	*p* (BMI)	Age β per year (95% CI)	*p* (Age)	Gender (Male vs Female) β	*p* (Gender)	KL grade (overall), F	*p* (KL)	Time (overall), F	*p* (Time)
KOOS4	- 0.948 (−1.541to −0.355)	0.002	- 0.273 (- 0.642, 0.097)	0.146	5.141	0.058	0.879	0.454	19.679	< 0.001
KOOS Pain	- 0.985 (−1.653 to −0.317)	0.004	- 0.370 (- 0.786, 0.046)	0.081	5.082	0.095	1.002	0.395	15.201	< 0.001
KOOS Symptoms	- 0.728 (−1.367 to −0.089)	0.026	- 0.058 (- 0.455, 0.340)	0.774	3.011	0.300	0.293	0.830	10.011	< 0.001
KOOS ADL	−1.018 (−1.640 to − 0.396)	0.002	- 0.393 (- 0.780, − 0.005)	0.047	6.558	0.022	1.549	0.206	21.037	< 0.001
KOOS QoL	−1.081 (−1.801 to − 0.362)	0.004	- 0.268 (- 0.715, 0.180)	0.239	5.695	0.083	0.639	0.592	12.598	< 0.001
VAS (0–10; higher = worse)	0.055 (−0.008 to 0.119)	0.087	- 0.019 (- 0.058, 0.021)	0.349	−0.401	0.165	2.454	0.067	56.413	< 0.001

Models used REML with random intercepts for participant and Satterthwaite df; fixed effects: time (baseline, 3 months, 12 months), gender, KL grade, age, BMI. Positive β indicates higher scores with higher covariate value; for KOOS outcomes, higher is better; for VAS, higher is worse. Gender β compares male vs female (reference). KL grade p-values are Type III (overall)

Concomitant therapies, including rescue analgesic use and physiotherapy, were recorded during follow-up and are reported descriptively. No formal adjustment for changes in co-interventions was performed. Rescue medication use (standardized to *n* = 92). Among 12-month completers, use of as-needed analgesics (on PRN basis) declined from baseline to 12 months: tramadol from 16/92 (17.4%) to 11/92 (12.0%) (- 5.4 percentage points (pp);—31.0% relative), celecoxib from 10/92 (10.9%) to 7/92 (7.6%) (- 3.3 pp;—30.3% relative), and topical analgesics from 8/92 (8.7%) to 4/92 (4.3%) (- 4.4 pp;—50.6% relative). Physiotherapy use declined from 11/92 (12.0%) at baseline to 2/92 (2.2%) at 12 months (- 9.8 pp; −81.8% relative). These standardized reductions are directionally consistent with the sustained improvements observed in KOOS and VAS over 12 months.

Safety profile. No adverse events of special interest were observed. Two participants (1.8%) reported transient post‑injection knee pain within 24 h; symptoms resolved without intervention within ≤ 48 h. No septic arthritis, hypersensitivity, or systemic complications were recorded.

## Discussion

To our knowledge, this prospective cohort is among the first to report 12-month outcomes after a single injection of double cross-linked HMWHA (Crespine Gel Plus), addressing an evidence gap left by prior Crespine series that ended at 9 months and by much of the IAHA literature that emphasizes less than 6-month horizons. This study observed durable, clinically meaningful improvements in pain and function in knee OA patient post Crespine Gel Plus injection, consistent with the broader IAHA evidence for symptom relief, and extends the durability signal to one year in a real-world Southeast Asian cohort where arthroplasty is often deferred [7, 9, 17, 18, 29].

This study used KOOS4, the mean of Pain, Symptoms, ADL, and QoL as the primary endpoint on psychometric and methodological grounds. These domains capture patient-centred function and health status while minimizing the floor effects and structural missingness that frequently affect Sport/Rec in older, the lower-activity cohorts. This choice is consistent with published guidance and prior use of KOOS4 in published literatures [20, 21]. In this cohort, KOOS4 improved by around 8.6—8.8 points at 3 and 12 months and remained stable. These findings are consistent with prior study in a cohort treated with high-concentration IAHA, which reported KOOS improvements sustained to 12 months and beyond [26], whereas a recent prospective study of cross-linked HMWHA demonstrated strong gains at 3–6 months with attenuation by 12 months, suggesting durability may be formulation and regimen dependent [30]. Systematic reviews focused on cross-linked HA also note symptom benefits detectable up to 12 months, supporting the plausibility of year-long persistence in some settings [15]. Against this backdrop, the maintenance of KOOS4 and KOOS sub scores gains to 12 months in this study adds real-world evidence of one-year durability with a single double-cross-linked HMWHA Crespine Gel Plus injection, while underscoring that longer-term trajectories may vary by formulation and retreatment strategy.

In the broader landscape of orthobiologic interventions for musculoskeletal conditions, recent studies provide complementary insights. A prospective observational study by Tripathi et al. reported that intra-articular autologous conditioned plasma was associated with significant reductions in pain and improvements in stiffness at 6-month follow-up in patients with knee osteoarthritis, suggesting that biologically derived injections may confer symptomatic benefit across different degenerative joint phenotypes [31]. Likewise, evidence from hyaluronic acid use in rotator cuff pathology indicates that HA injections can lead to improvements in pain intensity and functional scores compared with physical therapy and platelet-rich plasma in short-term follow-up, although differences versus placebo and corticosteroids were often not significant, and long-term durability remains less well established [32]. Together with our findings on cross-linked high-molecular-weight HA in knee osteoarthritis, these studies suggest that inert and biologically active injectable orthobiologics may provide symptom relief and functional gains in a range of joint pathologies. However, differences in mechanisms of action, tissue environment, and study design highlight the need for cautious interpretation and direct comparative trials to delineate relative efficacy, safety, and durability across orthobiologic modalities.

In this study, KOOS–QoL exhibited an incremental gain between 3 and 12 months. The delayed QoL improvement suggests that reductions in pain and functional limitation require time to translate into measurable gains in perceived life impact (for instance, activity resumption, confidence, social participation). This results is consistent with previous published literature showing significant QoL improvements by 3 to 6 months, often meeting MCID thresholds, with benefits sustained through 6 to 9 months [33, 34]. QoL is one of the core patient-centred outcome in hip and knee OA as it captures disease impact beyond pain and function. Accordingly, the OMERACT OARSI consensus designates QoL as a mandatory domain for measurement and reporting in all trials. QoL encompasses role participation, emotional well-being, and social or occupational functioning, and is responsive to clinically important change with established KOOS-QoL MCID thresholds. Its routine inclusion enhances cross-trial comparability, informs benefit–risk assessments aligned with patient priorities, and complements pain and function endpoints in defining overall treatment value [20, 21, 35]. For KOOS-QoL, the overall improvement from baseline to 12 months exceeded MCID thresholds, whereas the incremental gain between follow-up visits primarily reflects durability rather than additional clinical improvement. In the context of knee osteoarthritis, where symptom progression is expected, maintenance of benefit over time represents a favourable and clinically meaningful outcome. Interpretation of clinical relevance should distinguish statistical significance from patient-perceived benefit. In this study, pain relief assessed by VAS was maintained between 3 and 12 months without significant additional change, supporting sustained symptom control.

This study reported that each1 kg/m^2^ increase in BMI was associated with around 1-point lower KOOS, this pattern is consistent with published literatures which identified lower/normal BMI as a predictor of better response to IAHA injections, whereas obesity is repeatedly linked to weaker or shorter-lived benefit [36, 37]. Mechanistically, greater adiposity increases mechanical load across the knee and is linked to low grade inflammation and synovitis, the processes associated with worse patient-reported outcomes, including KOOS domains [38–40]. Studies indicate that higher body weight shortens the therapeutic window of IAHA; whereas overweight and obesity are associated with poorer responses and reduced durability of IAHA effect [41, 42]. These patterns align with guidelines that prioritize weight management alongside exercise to enhance and sustain outcomes in knee OA [43]. Thus, structured weight-loss counselling with referral to evidence-based programmes where appropriate should be incorporated into routine care in knee OA patients to achieve better outcomes.

There was a reduction in rescue-analgesic use at 12 months among subjects who completed 12-month follow up, this finding was consistent with prior studies showing that IAHA injection is often accompanied by decreases in NSAID/opioid consumption and corticosteroid injections [44–46]. Given the well-documented risks of gastrointestinal bleeding and increased cardiovascular events with chronic NSAID therapy in older adults, even modest reductions in rescue medication may give meaningful safety benefits [47, 48]. This study recorded no serious adverse events and only 2 reported cases of transient post-injection pain resolving within 48 h. The finding in line with most meta-analytic data which suggest that IAHA has a low adverse-event burden, most often short-lived local pain or swelling [49, 50]. Changes in concomitant therapies, such as rescue analgesic use and physiotherapy, may have contributed to the observed improvements and represent a potential source of confounding. As these co-interventions were not standardized and were analysed descriptively, their independent effects could not be disentangled from the observed outcome changes. Future studies should prospectively and systematically capture changes in concomitant therapies, including dose, frequency, and duration of analgesic use and physiotherapy exposure. Incorporating controlled measurement of co-interventions or randomized comparator designs would allow more robust attribution of observed effects to the intervention of interest.

This study is limited by its single-arm, open-label, single-centre design, which precludes causal inference and leaves findings vulnerable to expectancy/placebo effects and unmeasured confounding (e.g., activity modification, independent changes in analgesic regimens). In the absence of a comparator group, the results should be interpreted as associative rather than definitive evidence of treatment efficacy. The study population was predominantly composed of patients with KL grade II & III osteoarthritis, which may limit generalizability to patients with more advanced disease (KL grade IV) or to populations with different demographic or healthcare characteristics. Generalizability is also restricted by the Malaysian hospital setting, the single product evaluated, and a single-injection regimen; thus, extrapolation to other formulations, dosing schedules, or health-system contexts should be undertaken cautiously. This study relied predominantly on patient-reported outcome measures (KOOS and VAS) without the inclusion of objective functional or structural assessments. Although patient-reported outcomes are recommended core domains for knee osteoarthritis and capture clinically meaningful aspects of pain, function, and quality of life, the absence of performance-based measures or imaging biomarkers limits mechanistic interpretation and responder profiling. Future studies combining patient-reported outcomes with objective functional testing and imaging measures may offer a more comprehensive understanding of treatment response and durability. Lastly, although linear mixed-effects models are robust to missing data under the MAR assumption, attrition between the 3- and 12-month follow-up may still introduce bias if missingness was related to unmeasured outcome severity. Formal sensitivity analyses were not performed, and this should be considered when interpreting the long-term findings.

## Conclusion

A single intra-articular Crespine Gel Plus injection was associated with durable improvements across pain and function through one year and a favourable safety profile. Crespine Gel Plus may be considered a non-surgical option providing year-long symptomatic relief for patients with knee OA.

## Data Availability

The datasets collected and/or analyzed during the current study are accessible from the corresponding author upon reasonable request.

## Abbreviations

OA: Osteoarthritis
HA: Hyaluronic Acid
IAHA: Intra-Articular Hyaluronic Acid
HMWHA: High-Molecular-Weight Hyaluronic Acid
LMWHA: Low-Molecular-Weight Hyaluronic Acid
TKR: Total Knee Replacement
HSAH: Sultan Abdul Halim Hospital
MREC: Medical Research and Ethics Committee
MOH: Ministry of Health (Malaysia)
NMRR: National Medical Research Register (Malaysia)
KL Grade: Kellgren–Lawrence Grade
PRN: Pro Re Nata (as needed)
NSAID: Non-Steroidal Anti-Inflammatory Drug |
QoL: Quality of Life
ADL: Activities of Daily Living
Sport/Rec: Sport and Recreation Function
OMERACT: Outcome Measures in Rheumatology
OARSI: Osteoarthritis Research Society International
EULAR: European Alliance of Associations for Rheumatology
BMI: Body Mass Index
